# The concept of vulnerability and its relation to equality in the context of human rights: cases from climate change, anti-discrimination and asylum

**DOI:** 10.3389/fsoc.2025.1522402

**Published:** 2025-02-11

**Authors:** Monika Mayrhofer, Margit Ammer, Katrin Wladasch

**Affiliations:** ^1^Institute of Applied Sociology of Law and Criminology, Universität Innsbruck, Vienna, Austria; ^2^Ludwig Boltzmann Institute of Fundamental and Human Rights, Vienna, Austria

**Keywords:** vulnerability, equality, non-discrimination, human rights, climate change, asylum

## Abstract

The article analyzes the concept of vulnerability and its relation to the principles of equality and non-discrimination based on a theoretical discussion and the analysis of the concept in three case studies in different human rights areas. First, an overview of the development of the legal norms of the right to equality and non-discrimination in the context of social and political developments is given, including contextualizing and embedding the concept of vulnerability in this debate. Second, the results of three case studies from different policy and legal fields will be presented. The first case study focuses on the role of vulnerability in UN human rights documents on climate change and mobility, the second case study on the role of vulnerability in the anti-discrimination case law of European courts, and the third case study on the concept of vulnerability in Austrian asylum procedures. The objective is to study the concept in different settings and, subsequently, comparatively carve out common themes across the case studies. The case studies show that vulnerability is a fuzzy concept, which often ends up being attached to ‘special-needs groups’ and which frequently mobilizes stigmatizing and stereotyping narratives. The concept does not have equality-promoting connotations, emphasizes individual and group-specific deficit accounts, and often fails to grasp structural factors of discrimination and inequality.

## 1 Introduction

Vulnerability is a widely used concept among human rights scholars, in human rights policies and in human rights jurisprudence. The usage of the concept is, very often implicitly, assumed to enhance the protection of human rights, especially with regard to the principles of equality and non-discrimination. There has been some academic discussion on the substance, implications and application of the concept from a human rights perspective and whether it promotes or hampers equality and non-discrimination (Brown, 2011; Fineman, 2008, 2017; Scully, 2009; Nifosi-Sutton, 2017; Ippolito and Iglesias Sanchez, 2015; Peroni and Timmer, 2013; Mayrhofer, 2020). Some refer to the potential promises of the concept such as being a more substantial basis for equality. As vulnerability is claimed to be ‘natural and inevitable’ (Fineman, 2016, p. 17), the vulnerable subject is proposed to be a more universal figure than the liberal subject. In addition, the recognition of vulnerability is perceived as a ‘condition for the respect of human dignity’ (Masferrer and García-Sánchez, 2016, p. 1); the concept is assumed to allow for getting rid of identity categories and replacing the norm of the liberal subject with the ‘vulnerable’ subject. In doing so, it is assumed to overcome the limits of formal equality, which is based on the ideal conception of the liberal, independent and rational subject, and to concentrate on the structures of society (Grear, 2016, p. 41). More skeptical researchers, however, point out that the term is deficit-oriented and leads to stigmatization (Brown, 2011, p. 319; Urquiza Haas and Sánchez García, 2015, p. 151). It tends to accentuate people’s weaknesses and limitations, and runs the risk of portraying people as passive and unable of bringing about change (Wisner, 2004, p. 13). They criticize the vagueness of the concept, which undermines ‘its promise as a conceptual frame to understand and challenge systematic inequalities’ (Cole, 2016, p. 266) and makes it ‘difficult to reconcile with ensuring equal protection of human rights’ (Heri, 2021, p. 205). Furthermore, critical scholars highlight the reduction of the concept to specific ‘vulnerable groups’, which is often not only a stereotypical representation of these groups but may also hamper human rights objectives. For example, the labeling of specific groups as vulnerable may have negative consequences for groups or individuals excluded from the concept (Scully, 2009) or may be complicit in practices of essentialism, stigmatization, and paternalism (Peroni and Timmer, 2013; Brown, 2017; Kadetz and Mock, 2018, p. 215). It was also argued that the concept is a problematic entry point into politics as the ‘vulnerable citizen is in certain respects the antithesis of proper citizenship’ (Brown, 2017, p. 670). In addition, it was pointed out that as a result of vulnerability reasoning, ‘dynamics of dominance and inequality shift towards questions of feeling’ (Cole, 2016, p. 274) and that ‘vulnerability embodies the absence of […] power […]; it is an obstacle to freedom; and it prevents equality’ (Ferrarese, 2016, pp. 155–156).

This article aims to contribute to the analysis of the relationship, complementarities, overlaps, ambiguities, delimitations or even inconsistencies of vulnerability with the concepts of equality and non-discrimination by comparatively presenting the results of three case studies in three different human rights areas where the concept of vulnerability is used. Case studies were chosen in fields where the researchers had observed for some time that the concept of vulnerability is used and applied rather extensively, and where questions of inequality and discrimination also play an essential role. Thus, the case studies were chosen purposively, which means that the researchers decided to select ‘information-rich cases’ from which researchers ‘can learn a great deal about issues of central importance to the purpose of the inquiry’ (Flick, 2022, p. 1173). In addition, it was intended to choose very different cases^1^ as the objective was to compare ‘instances of the same phenomenon in different circumstances’ (Bleijenbergh, 2010, p. 555). The selected case studies represent cases from the global (UN), regional (European), and national (Austrian) levels. The first case study is located in the field of climate change-related mobility and analyses how the concept of vulnerability is used and applied in UN human rights documents focusing on different dimensions of (im)mobility in the context of climate change. The second case study was selected in the field of anti-discrimination and equality law and analyzes the concept of vulnerability in anti-discrimination case law of two European Courts, the European Court of Human Rights (ECtHR) and the Court of Justice of the European Union (CJEU). The third case study concentrates on the field of asylum and elaborates on how the concept of vulnerability is used in case law relating to international protection at the Austrian level. Thus, the objective is to study the concept of vulnerability in different settings (case studies) and carve out common themes and patterns that emerge concerning the concepts of equality and non-discrimination. This is based on an understanding of equality and non-discrimination not only as a distinct policy field—hence, the choice of a case study in this field—but also as a cross-cutting issue. Thus, we aim to compare equality-related themes that are relevant in all three case studies. The comparative analysis aims to contribute to the vulnerability debate from the perspective of equality and non-discrimination, as the latter are core principles of the international and regional human rights framework, which have been developed and refined in different phases (see Section 2).

The analysis will focus on whether the concept of vulnerability is defined in the material, which was sampled and analyzed during the case studies, and if so, how the concept is defined and whether and how this definition differs from definitions of equality and discrimination. Furthermore, the objectives, motivations and legitimizations for the introduction and use of the concept are examined, also in terms of whether they are related to inequality and discrimination. In addition, it is analyzed whether the material sheds light on how it conceptualizes vulnerability in relation to the concepts of (in)equality and discrimination. That means, it mainly applies inductive reasoning to find out how vulnerability is differing from, contradicting or overlapping with the concepts of (in)equality and discrimination. This is based on the understanding that the choice of concepts is significant in the legal and political discourse as they have an impact on how we frame the issues at stake. Concepts are essential for how ‘we make sense of the social world. They are labels that we give to aspects of the social world that seem to have common features that strike us significant’ (Clark, et al., 2021, pp. 8–9). Lakoff and Johnson stress the importance of metaphors for the human conceptual system. ‘The essence of metaphor is understanding and experiencing one kind of thing in terms of another’ (Lakoff and Johnson, 2003, p. 9). These metaphorical concepts have powerful consequences as they have ‘the power to define reality. They do this through a coherent network of entailments that highlight some features of reality and hide others’ (ibid, p. 6). Thus, a specific emphasis in this article is laid on the narratives and stories associated with the concept of vulnerability. In the legal and political context vulnerability functions as a somatic metaphor, as vulnerability literally means wound or the possibility to be wounded, which is transferred to political and legal contexts. It will be analyzed how the narratives and stories associated with the concept of vulnerability relate to equality and non-discrimination. In doing so, the analysis aims to give insight into the role of the concept of vulnerability with regard to the containment of discrimination and the enhancement of equality.

The case studies applied a qualitative-interpretive design^2^ and used different forms of texts as the primary source of data. Texts were defined in a broad manner comprising a wide variety of different sources including texts produced in a policy context (such as strategy papers, policy papers, action plans, policy reports and other documents), legal texts (such as laws, jurisprudence and other juridical instruments), or narrative texts that were obtained by semi-structured interviews with different stakeholders. The research, therefore, mainly analyzed already existing texts (such as policy and legal documents) but also texts produced during the research process (interviews). The texts/interview partners in the different fields were ‘sampled purposively’ on the basis of being particularly relevant and informative concerning the topic of interest (Flick, 2014, pp. 170–174; Lindekilde, 2014, p. 211). The sampling was done as a step-by-step process: Texts and interview partners were selected ‘according to their (expected) level of new insights (…). Sampling decisions aim at the material that promises the greatest insights, viewed in light of the material already used, and the knowledge drawn from it’ (Flick, 2014, p. 171). Sampling was limited on the one hand by the specific thematic field or case study or when the criterion of ‘theoretical saturation’ was reached (Flick, 2014, p. 172). Thus, the process of purposive sampling resulted in the selection of different types of texts for each case study, as only texts that seemed particularly relevant were selected for each case study. The data was first structured (thematic coding), i.e., the data was labeled and categorized according to a ‘thematic framework’,^3^ before it was interpreted (Flick, 2014, pp. 420–427; Spencer et al., 2014, pp. 282–298). Some codes were defined in advance (deductive coding) according to the theoretical framework (see Section 2) as well as the research focus but the research design was flexible enough to incorporate ‘what the data tells us on its own’ (inductive coding) (Lindekilde, 2014, p. 213). In a last step, cross-case study themes that were particularly relevant to issues of equality were developed and codes were grouped and analyzed according to these themes.

The paper is structured as follows: In a first step, an overview of the development of the main concepts and points of discussion on equality and (non-)discrimination is given, including contextualizing and embedding the concept of vulnerability in this debate. Subsequently, the results of each case study are presented individually. The last section presents a comparative discussion of the overarching findings of the case studies including conclusions and possible ways forward.

## 2 Vulnerability and equality/non-discrimination—similar or different concepts?

This section gives an overview of the historical evolvement and different conceptual dimensions of the rights to equality and non-discrimination. It presents different development phases of equality theory in which new questions on how to conceptually and theoretically grasp and address different forms of inequalities and discrimination were raised and in which new approaches and concepts were developed that now have a firm place in international as well as regional equality and anti-discrimination law.^4^ The section also discusses when the concept of vulnerability emerged in the academic and legal debate and how it is related to the concepts of equality and non-discrimination.

The right to equality and the prohibition of discrimination are fundamental principles of the international human rights system. Indeed, human rights are based on the idea that ‘all human beings are equal’ and have the ‘same set of fundamental rights’ (Moeckli, 2018, p. 148). Human rights instruments usually contain a prohibition of discrimination and a right to equality (Smith, 2012). Yet, the liberal and formal conceptualization of equality has been repeatedly criticized and challenged over time, amongst others by feminist activists and researchers (Lacey, 2004, p. 21; Parisi, 2010; Otto, 2018). The initial human rights focus on achieving formal equality, equal (legal) treatment and abolishing direct discrimination was extended by incorporating perspectives of difference and notions of substantive equality as well as indirect and structural forms of discrimination (see, for example, Moeckli, 2018, pp. 148–164; Otto, 2018, pp. 309–325; Fredman, 2016b; Lacey, 2004; Parisi, 2010; Squires, 2001; Fredman, 2011). The development reflects different dimensions of equality theory in academia and different phases of equality activism in political practice (Squires, 2001). The first dimension refers to the so-called ‘equality perspective’^5^ and assumes that formal equality which means guaranteeing everyone the same rights—e.g. granting women the same rights as men—and abolishing (direct) discrimination on certain grounds would also lead to an equal enjoyment of rights. Formal equality and the prohibition of direct discrimination is a cornerstone of international human rights law,^6^ as well as of European equality and non-discrimination law (see, e.g., Tobler, 2014; Fredman, 2016a; European Union Agency for Fundamental Rights et al., 2018; Council of Europe/European Court of Human Rights, 2022). Formal equality refers to the idea that individuals should be equal before the law and equally protected by the law. Direct discrimination is usually defined as ‘less favourable or detrimental treatment of an individual or group of individuals on the basis of a prohibited characteristic or ground such as race, sex or disability’ (Interrights, 2011, p. 18).

The assumption that formal equality alone would suffice to achieve equal exercise of rights came under increasing criticism. In the context of the second phase of equality theory (‘difference perspective’), it was criticized that ignoring differences through seemingly neutral treatment and laws led to the perpetuation of inequalities (Otto, 2018). It was also argued that differences should rather be considered as value (Squires, 2001, pp. 9–10) and, thus, should be embraced (Committee on the Rights of Persons with Disabilities, 2018, para. 10). Subsequently, a more encompassing definition of discrimination (in particular the concept of indirect discrimination, which considers the disproportional effects of seemingly neutral laws, policies and practices on prohibited grounds of discrimination, but also other forms of disadvantageous treatment) was developed and laid down in international and European equality and non-discrimination law.^7^

The prohibition and elimination of direct, indirect and other forms of discrimination are understood to be crucial for achieving substantive equality, which – alongside formal equality – is understood as an essential dimension of equality. Substantive equality ‘is concerned with the effects of laws, policies and practices and with ensuring that they do not maintain, but rather alleviate, the inherent disadvantage that particular groups experience’ (Committee on Economic, Social and Cultural Rights, 2005, para. 7). Substantive equality also ‘refers to the notion that individuals in different situations should be treated differently’ (Interrights, 2011, p. 17) and seeks ‘to address structural and indirect discrimination and takes into account power relations’ (Committee on the Rights of Persons with Disabilities, 2018, para. 10). Yet, the debate on what exactly (substantive) equality means and which aspects and dimensions the concept of non-discrimination encompasses is still ongoing. Scholars as well as relevant political and legal actors and bodies (e.g., UN treaty bodies) are continuously developing these concepts further (see, e.g., Fredman, 2023; Moeckli, 2018; Smith, 2014; Wouters and Ovádek, 2021).

Two points should be highlighted in this context as they will be important later when discussing the concept of vulnerability: Firstly, there is an understanding that sexist, racist, ableist, age-based and other stereotypes, prejudices and stigma are incompatible with human rights obligations and they must be addressed in order to move toward the goal of substantive equality.^8^ As the European Commission against Racism and Intolerance (ECRI) has pointed out, this includes becoming aware and addressing processes of ‘racialization’ (ECRI, 2021). Racialization is a concept that has the potential ‘to aid understanding of the processes underpinning racism and racial discrimination’ (ECRI, 2021, para. 5). The concept ‘provides for an insight into the social and ideological processes that develop the stereotyping and reductive understanding of diverse human identities in racial terms as an exercise of power’ (ECRI, 2021, para. 3). Through a process of racialization ‘human populations (identified by, for example, phenotype or cultural identifiers) are ascribed […] certain characteristics and attributes that are presented as being innate to all members of each group concerned’ (ECRI, 2021, para. 3). Secondly, in order to achieve substantive equality, State Parties are allowed or even obliged under human rights and equality law to adopt targeted measures and policies to eliminate disadvantages against certain persons and groups or to specifically support these persons. The commitment to such proactive measures is based on the recognition that in order to achieve substantive equality a purely complaints-led model—which in case of discrimination requires an individual person to lodge a complaint at a court or another competent body—is important but not sufficient (Fredman, 2009, p. 1). As discriminatory attitudes and structures are often deeply embedded in society, proactive measures aim at systematic change and address institutional and structural forms of inequality (Fredman, 2009, p. 3). In order not to be considered unlawful discrimination, such proactive measures have to comply with well-defined criteria.^9^

Moreover, in recent decades the concepts of equality and non-discrimination have been challenged and advanced by approaches of diversity and intersectionality (Crenshaw, 1989, 1991; McCall, 2005; Carbado et al., 2013; Sigle-Rushton and Lindström, 2013; Mayrhofer, 2021; Squires, 2001). This reflects the third phase of equality theory, which was also called a ‘diversity perspective’ (Squires, 2001, pp. 12–13). Common to these approaches is the discomfort with and critique of monolithic conceptions of identity categories, such as the understanding of women as a homogeneous group with shared problems and similar concerns and interests and the disregard of differences, pluralism and complexity within a particular ‘group’. A meanwhile well-known and wide-spread concept, the concept of intersectionality, has been coined in this context.

The concept of vulnerability can also be located in this third equality phase, in which criticism of monolithic conceptualizations of identity- and group-based equality and non-discrimination categories has been increasingly raised. Vulnerability is suggested to be ‘a critique of dominant modes of thinking about inequality that is at once complementary to but more powerful than dependency’ (Fineman, 2008, p. 11). In particular, Martha Fineman’s theory of vulnerability has influenced many human rights scholars and still is one of the widely-used approaches in this context, especially among legal scholars. Fineman criticizes a concept of equality, which is closely linked with a non-discrimination approach (Fineman, 2008, p. 2). She points out that ‘under both the vulnerability and nondiscrimination approach the mandate is the same—the establishment of a regime of equality—but the foci and indeed the manner in which equality is imagined are very different’ (Fineman, 2008, p. 20). She criticizes that the equal treatment model relies on the myth of the autonomous individual and ‘fails to address substantive inequalities and differential allocations of privilege produced by our institutions’ (Fineman, 2008, p. 19). She suggests to place the ‘vulnerable subject […] at the center of our political and theoretical endeavors’ as it is more reflective of the actual human experience (Fineman, 2008, pp. 1–2). Such an approach would ‘recognise the ways in which power and privilege are conferred through the operation of societal institutions, relationships and the creation of social identities, sometimes inequitably’ (Fineman, 2017, p. 142). Furthermore, Fineman points out that the predominant equality/non-discrimination approach restricts the prohibition of discrimination to a few groups within society and does not outlaw discrimination in general (Fineman, 2016, p. 14). She also rejects the notion of intersectionality, which she sees as an expansion of ‘traditional equal protection analysis to account for multiple intersecting identities’ (Fineman, 2008, p. 15). Instead, the vulnerability thesis as conceptualized by Fineman is suggested to provide for a ‘post-identity’ inquiry which goes ‘beyond the stifling confines of current discrimination-based models toward a more substantive vision of equality’ (Fineman, 2008, p. 1). As vulnerability is understood to be a universal condition it is also supposed to transcend historic categories of discrimination (Fineman, 2008, p. 16). One of the main advantages of the vulnerability thesis, according to Fineman, is the assumption that a ‘vulnerability analysis concentrates on the structures our society has and will establish to manage our common vulnerabilities’ (Fineman, 2008, p. 1). She claims that a vulnerability approach suggests a more thorough and pervasive equality analysis and takes account of structural and institutional arrangements when appraising the response of the state to situations of vulnerability before charging the individual (Fineman, 2008, p. 17).

As already indicated at the beginning, many scholars have also criticized the concept and pointed out that it might have problematic consequences for addressing inequality and discrimination. The most important critical points are, firstly, the broadness, fuzziness and elusiveness of the concept (Brown et al., 2017, p. 505; Chapman and Carbonetti, 2011, p. 723) and, thus, its incapability of exactly grasping the complexity of structures and different forms of inequalities (Cole, 2016, p. 266). Secondly, vulnerability approaches have been accused of not being able to grasp power and political structures, which are closely interwoven with questions of inequality and discrimination. Instead, they are ‘invested in presenting vulnerability as being foremost universal, always ambivalent and ambiguous, at a distance from questions of power and politics’ (Cole, 2016, p. 267). Thirdly, although vulnerability is proposed to be a universal ‘post-identity’ category, it all the more ends up being frequently attached to certain groups (e.g., women, children, migrants). The concept of vulnerability has problematic repercussions for people included in the concept, as whenever vulnerability is applied only ‘to “marginal” subjectivities and exceptional situations, ideologies about the body as a naturally-given are reified, effacing the deeply political, exclusionary, and gendered and cultural affiliations’ (Urquiza Haas and Sánchez García, 2015, p. 152). The concept of vulnerability has also problematic consequences for people excluded from the concept, as even Fineman critically points out, as ‘designating only some as constituting vulnerable subpopulations is that such a designation suggests that some of us are not vulnerable’ (Fineman, 2016, p. 16). In addition, and fourthly, vulnerability is frequently criticized as being a concept that is essentializing and stigmatizing to those associated with the concept and reinforcing stereotypic representations of specific individuals and groups, ‘implying deviation from usually undefined standards of life or behaviour, and as supporting powerful moral and ethical projects’ (Brown et al., 2017, p. 498). The vulnerability concept is not necessarily associated with having equal rights but rather with providing aid and social benefits to those who are vulnerable and, thus, have special needs and ‘deserve’ support and aid (Smith and Waite, 2018, p. 1; Howden and Kodalak, 2018; Yahyaoui Krivenko, 2022). Indeed, the concept has been accused of preventing equality (Ferrarese, 2016, pp. 155–156). In addition, the label of vulnerability does not have particularly empowering associations, it rather implies a state of weakness (Brown, 2011, p. 314). The concept is associated with being dependent on others, being inactive and in danger, being restricted, powerless and needing help. The vulnerable person is defined as the problem, as being in need of special protection: Vulnerable individuals are framed as ‘a problem to be addressed’ (Brown, 2017, p. 670). Closely related with the previous point is the issue that this ‘approach can reduce people to being passive recipients, even “victims”’ (Wisner, 2004, p. 13). Finally, it has also been pointed out that vulnerability is problematic with regard to politics (Cole, 2016, p. 273) as vulnerability is not necessarily a claim about injustice nor a claim that indicates what is wrong. The concept of vulnerability is also criticized for failing to tackle structural factors of inequality and disadvantage as narratives of vulnerability are said to reinforce individual deficit accounts of disadvantage and adversity rather than focusing on structural factors (Brown, 2017, pp. 674–675). The associations of vulnerability as an ‘ontological condition’ (Turner, 2006, p. 9), as something which is given and something we cannot ‘argue against’ (Butler, 2004, p. 19) means disassociating it with politics and marking the issues associated with vulnerability being beyond political influence (Mayrhofer, 2020, p. 160). The vulnerability approach is also criticized as being a particular troublesome approach in the context of being recognized as equal subjects in the political sphere. Often, the calls on the need of vulnerable persons to be protected ‘have sat uneasily with the full inclusion’ of these persons ‘in the political sphere’ (Scully, 2009, p. 119).

In order to elaborate on whether the concept of vulnerability lives up to the promises indicated above or whether, in the contrary, has problematic consequences for the enhancement of equality, the results of three case studies will be presented. After outlining the background to and the material taken into account for the respective case, it will be analyzed whether and how the concept is defined in the particular case, what are the (legal or other) sources of vulnerability and whether the cases reveal anything on the objectives of applying the concept. It will further be discussed who is framed as vulnerable in the respective case and whether this framing relies on ‘traditional’ identity categories. Moreover, the case studies will analyze the narratives mobilized by the vulnerability concept and whether these narratives reinforce stereotypic representations of specific individuals and groups. A particular focus of the case studies will be laid on the discussion of the questions how vulnerability in the respective case is interrelated with or differs from equality and non-discrimination and whether conclusions can be drawn as to whether this promotes equality.

## 3 Case study 1: Vulnerability in UN human rights documents on climate change and mobility

Since more than 15 years, UN human rights bodies and mechanisms have increasingly discussed the relationship between climate change and human rights, including human rights challenges of different forms of climate change-related mobility. They have adopted many resolutions and published several reports and other documents focusing on climate change and human rights in general and climate change, human rights and mobility in particular. Thus, the 68 documents that were selected for analysis while conducting this case study are reports, policy briefs or documents, case law, declarations, resolutions, recommendations or comments, which focus either entirely on human rights issues and state obligations in the context of climate change and different forms of mobility or which address the issue partly in a substantive way^10^ and which were published by the UN Human Rights Council (HRC), special procedure mechanisms, OHCHR, but also by treaty-based human rights bodies.

### 3.1 Legal sources, definition, and objectives

The concept of vulnerability is widely used in the analyzed documents although no legally binding international human rights instrument the documents refer to contain a reference to vulnerability. Throughout the documents, vulnerability is predominantly used without providing a (clear-cut) definition of or stating the objectives for using the concept. In documents where definitions of vulnerability are indicated—often not explicitly—, they tend to be very broad and general and, thus, usually fail to indicate the delimitations of the concept, and to provide an unambiguous understanding of vulnerability. An exception is the report *The Slow onset effects of climate change and human rights protection for cross-border migrants*, which explicitly defines the concept. The report adopts ‘an understanding of vulnerability that is focused on a person’s relative ability to effectively exercise their human rights.’ The report further points out that ‘[i]ncreased vulnerability also means that an individual is likely to have less adaptive capacity—or ability to adjust or respond to the impacts of climate change’ (UNHRC, 2018, para. 52). The definition indicates that individuals’ (in)ability to exercise their human rights or adjust to the impacts of climate change is understood as the problem and framed as their vulnerability. This is a considerable difference compared to definitions of direct or indirect discrimination which focus on the less favorable treatment of a person on specific grounds or the ‘distinction, exclusion, restriction or preference’ based on a specific ground which either has ‘the purpose or the effect of nullifying or impairing the recognition, enjoyment or exercise, on an equality footing, of all rights and freedoms’ (Human Rights Committee, 1989). The vulnerability definition indicates the affected individuals and their deficits as the problem (see also Section 3.2 on the narratives that are evoked in the documents and which correspond with this definition). In contrast, the definition of discrimination conceives the action and structures that have an adverse impact on individuals as the problem. This tendency of ‘individualizing’ social or political problems and risks [e.g., lack of resources (Jimenez-Damary, 2020, para. 15), barriers to international migration (UNHRC, 2018, para. 52), gender-based violence (Jimenez-Damary, 2020, para. 32)] by framing it as the vulnerability of individuals or groups of persons is apparent in all documents.

### 3.2 Application of the concept: Who is framed vulnerable? What are the narratives associated with vulnerability?

Vulnerability is often used to refer to specific groups and individuals. Increasingly, not the phrase ‘vulnerable groups’ or ‘individuals’ is used but phrases such as groups, persons or individuals ‘in situations of vulnerability’ or ‘in (a) vulnerable situation(s)’ (Crépeau, 2012; UNHRC, 2018; OHCHR, 2021). Persons and groups, frequently referred to as vulnerable in the context of climate mobility are women (and girls), children, migrants, refugees, disabled, older persons, indigenous people, the poor and minorities. Although there is a considerable variety of different (sub-)groups labeled as vulnerable, some groups are identified considerably more often to be vulnerable or in a vulnerable situation (in particular women and different sub-groups of women, children and the poor). Furthermore, it is not clear, what qualifies a certain group as being vulnerable or in a vulnerable situation. Thus, in the documents the vulnerability approach predominantly ends up being attached to certain pre-determined identity-based groups (e.g., women, children, migrants, the poor). In none of the documents, men and boys are explicitly referred to as vulnerable, which, from a gender perspective, reinforces a reductionist and stereotypical gender model. The same applies to other social categories where the vulnerability concept tends to perpetuate a binary identity- and group-based approach. From a ‘diversity perspective’ (see Section 2), this practice neglects the diversity and complexity of and between groups and, in particular, fails to conceptually grasp the role of ‘dominant’ groups.

The narrative frequently invoked in the documents in relation to the concept of vulnerability is that of lists of (potential or actual) harm, abuses and sufferings faced by vulnerable individuals and groups. They are described as those who ‘suffer the most’ (Kälin, 2009, para. 25). Vulnerability is a concept, which is associated with many problematic and adverse adjectives and situations. It stands for a broad range of adversities such as diseases, illnesses, hunger, death but also violence, conflict, exploitation and abuse. For example, it is pointed out that ‘[u]nfortunately migrants are facing increasing intolerance and are becoming more vulnerable to potential racist or xenophobic outbreaks of violence, or they may fall prey to criminal traffickers and smugglers’ (Bustamente, 2011, para. 25). Another example states that *‘*women and girls are particularly vulnerable to the adverse effects of climate change and at higher risk of violence during displacement’ (Jimenez-Damary, 2020, para. 32). Thus—and as also indicated above—, vulnerability is often phrased as something which is a failure or flaw of the specific person or group (‘women and girls are vulnerable to’). They are described as those, who ‘are entitled to protection and assistance required by their condition and to treatment which takes into account their special needs’ (Kälin, 2009, para. 31). It has been argued that human rights approaches often frame people moving in the context of climate change as vulnerable, helpless and passive victims who are in need of protection (Ransan-Cooper et al., 2015, p. 106; Oakes et al., 2020). The analysis of the documents suggests that the concept of vulnerability is an important part of this victim-protection narrative (Ransan-Cooper et al., 2015, p. 109). The ‘needs’ of vulnerable persons are often labeled as ‘special’ or ‘particular’, thus, implicitly referring to an invisible standard of those (male, wealthy, healthy, sedentary, middle-aged and able-bodied persons) who have ‘normal’ needs so that they are not even mentioned as needs at all. These problematic, stigmatizing and patronizing narratives of different forms of suffering, neediness, and particularities—in short ‘Otherness’—of those marked as vulnerable rely on an implicit understanding of ‘norm and deviation’ and suggest an inferiority of persons marked in such a way that is at odds with principles of equality and non-discrimination.^11^

### 3.3 Relationship between vulnerability and equality/non-discrimination

The documents also explicitly relate inequality and discrimination to vulnerability. They frequently emphasize that inequality and discrimination are understood as factors that lead to more vulnerability. For example, a Report of the OHCHR published in 2018 says that vulnerability ‘can result from multiple and intersecting forms of discrimination, inequality and structural and societal dynamics that lead to diminished and unequal levels of power and enjoyment of rights’ (UNHCHR, 2018, para. 14). A direct causality between discrimination/inequality and vulnerability is established on many occasions. However, the relationship is also described vice versa (UNHRC, 2018) or even as a spiral. That means, vulnerability is not used as a synonym for inequality or discrimination, but rather as a distinct concept, which is used to indicate all the problematic effects (harm, risks, health risks, violence) which result *inter alia* from discrimination and inequality and which further creates and reinforces inequality and discrimination. Vulnerability therefore is understood as a problematic issue with regard to achieving equality and abolishing discrimination. In the documents, it is frequently indicated that vulnerable persons or groups should be given ‘special protection’ or ‘priority treatment’. For example, it is emphasized that resettlement opportunities should ‘be offered in a non-discriminatory manner, with priority being given only on the basis of specific vulnerability or need’ (Kälin, 2009, para. 41). However, due to a lack of clarity of criteria who is counted vulnerable there is the risk of stereotypical or arbitrary application, which is at odds with principles of equality and non-discrimination.

To sum up, the documents analyzed for this case study usually neither define vulnerability nor do they indicate the added value of this label. In the rare occasions, when a definition is provided, it emphasizes the lack of ability and deficiencies of a person or of groups. The documents usually list and discuss a broad range of problems and challenges associated with different forms of climate mobility and frequently frame these problems and challenges as the vulnerability of certain individuals, groups and communities. The concept tends to perpetuate binary identity- and group-based approaches. The narrative mobilized in the documents in relation to vulnerability is a broad range of harm, abuses, risk and sufferings that are associated with these groups. They are stereotypically presented as those who have special needs and should receive particular protection and priority treatment and thus rely on an implicit understanding of an unspoken norm (white, male, middle-aged, wealthy, sedentary, able-bodied, healthy) and its deviation (female, migrants, disabled, poor, elderly, children, ill). Inequality and discrimination are frequently understood as factors that lead to vulnerability and vulnerability, in turn, is often understood to result in inequality and discrimination.

## 4 Case study 2: The role of vulnerability in the anti-discrimination case law of European courts

The starting point for investigating the application of the concept of vulnerability in anti-discrimination case law of European courts [the ECtHR (Council of Europe (CoE)] and the CJEU [European Union (EU)] was the observation that it is utilized inconsistently at the European level (EU and CoE). The concept of vulnerability is used in the case law of the ECtHR, but not in that of the ECJ. Furthermore, the application of the concept by the ECtHR was repeatedly criticized by scholars for being ambiguous and incoherent and for contributing to essentialism, stigmatization, and paternalism (Kim, 2021; Peroni and Timmer, 2013). Nevertheless, the concept continues to be used by the ECtHR. The data analyzed for this case study was, on the one hand, obtained by seven interviews conducted with members/representatives of the EU, the European Committee of Social Rights (ECSR), Equinet, and the Council of Europe (CoE).^12^ The objective of the interviews was to determine the objectives, motivations, and legitimation for introducing and using the concept. On the other hand, ECtHR case law, which uses the concept of vulnerability explicitly in discrimination claims (Art. 14 ECHR),^13^ was selected and compared to case law by the ECJ that concerns similar subject matter, yet does not use the concept of vulnerability. The relevant case law of the ECtHR and the CJEU in the field of non-discrimination was analyzed to determine whether and how the concept of vulnerability is applied. As the concept of vulnerability is criticized by scholars for reinforcing stereotypes, prejudice and stigma rather than acknowledging the need to address structural patterns of discrimination (for a detailed overview and sources of the academic debate, see Section 2), this case study focuses in particular on how the two courts approach this issue in their non-discrimination case law.

### 4.1 Legal sources, definitions, objectives and motivations for introducing and using the concept

Vulnerability is not defined or mentioned in the relevant legal framework, which comprises the European Convention of Human Rights (ECHR),^14^ the Charter of Fundamental Rights of the European Union (CFREU), and the EU anti-discrimination Directives. The first traces for the origin of the usage of the concept that could be established were linked to the case law of the ECtHR and to recommendations by the European Commission against Racism and Intolerance (ECRI) (ECRI, 1998) and the European Committee of Social Rights (ECSR). Several interviews with representatives of the ECSR, the European Network of Equality Bodies (Equinet), the CoE and the EU confirmed that the concept was increasingly used. The reasons assumed by the interview partners for introducing and using the concept ranged from the need to identify those who need the most protection or specific attention to pragmatic arguments that the concept helps explain a need for action to policymakers and motivates them to give funds. Furthermore, it was argued, that the ECtHR shows a certain hesitance towards using the concept of structural discrimination. This would make the application of the concept of vulnerability strategically important as it helps to address structural factors without necessarily naming them as such. Most of the interviewees, however, also voiced the concern that declaring specific groups as a whole as vulnerable is accompanied by the risk of stereotyping and the danger that protection is provided only to selected groups. Yet, there was no clear explanation of why the concept is used in some circumstances and not in others.

### 4.2 Application of the concept: Who is framed as vulnerable? What are the narratives associated with vulnerability?

There is a large number of ECtHR case law in which discrimination is claimed in addition to the violation of a right or freedom guaranteed by the Convention (ECRI, 1998). The Court, however, often stopped examining the discriminatory aspect of the case once it had found a violation of the right or freedom in question. Only in recent years, it increasingly recognizes and explicitly addresses discriminatory aspects of human rights violations. In such cases, the Court more and more frequently refers to the concepts of direct and indirect discrimination and reasonable accommodation developed at the EU level (i.e., EU law and CJEU case law). At the same time, it also applies narratives of vulnerability in the reasoning of such cases. This mostly includes Roma (D.H. and Others v. the Czech Republic, 2007; Oršuš and Others v. Croatia, 2010) and disability (Alajos Kiss v. Hungary, 2010; Konstantin Markin v. Russia, 2012; Çam v. Turkey, 2016) rights cases. Yet, it is not clear, why this reference to vulnerability is made. The Court in *D.H. & Others v. the Czech Republic*, for example, provides a profound analysis of the selection procedure for pupils who are to attend special schools. Based on statistical data, which show a disproportional number of Roma children in special schools, and expert statements, which demonstrate that the tests applied were designed according to the norm of a Czech child that is raised with Czech culture and language, the Court finds indirect discrimination. Repeatedly in the very same case, it also refers to the history of Roma segregation in education and their vulnerable position in society, concluding that ‘special consideration should be given to their needs and their different lifestyle’—but why this reference is made is not explained. The Court has confirmed and extended this approach towards Roma and the specific attention they should be given in consecutive judgements, like for example in Oršuš v. Croatia, where it states that ‘(…) as a result of their history, the Roma have become a specific type of disadvantaged group and vulnerable minority (…). They therefore require special protection’ (D.H. and Others v. the Czech Republic, 2007). In what way this acknowledgement is influencing the decision on discrimination is not explained, the Court simply states that it ‘cannot ignore that the applicants are members of the Roma minority’ and that ‘therefore, in its further analysis the Court shall take into account the specific position of the Roma population’ (Oršuš and Others v. Croatia, 2010). It is not explained why the reference to vulnerability was relevant to the finding of indirect discrimination.

A focus on the specific situation or needs of certain groups is also characteristic of disability rights cases. The ‘particular vulnerability’ of children with disabilities is mentioned by the Court as an argument for specific attention in making choices on how their needs should be accommodated (Enver Şahin v. Turkey, 2004; Çam v. Turkey, 2016) and not for the finding of discrimination. The Court for its discrimination analysis argues that Article 14 has to be interpreted in the light of the requirements set out by the UN Convention of Persons with Disabilities (UNCPRD), meaning that a denial of reasonable accommodation measures has to be considered as discrimination. All these cases adopt a stereotyping approach towards groups of people labeled as vulnerable, connecting minority status and historical injustice with a situation of deprivation and disability with an automatic need for specific attention.

Some cases also include a reference to the vulnerable situation of women (Opuz v. Turkey, 2009; Khamtokhu and Aksenchik v. Russia, 2014), but there is a little bit more hesitance due to the acknowledgment that ‘differences based on sex require particularly serious reasons by way of justification and that references to traditions, general assumptions or prevailing social attitudes in a particular country cannot, by themselves, be considered to amount to sufficient justification for a difference in treatment’ (Khamtokhu and Aksenchik v. Russia, 2014, para. 78). The Court could have limited its analysis to statistics indicating an unequal position of women compared to men, in order to argue that this ‘required action on the part of authorities in order to redress the disadvantage’ (A.E. v. Bulgaria, 2023). Instead, parallel to the discrimination analysis, it continues to refer to the special vulnerability of women.

The narratives associated with or supported by the vulnerability concept in these cases (either by the Court, by legal representatives, or by State Parties) are that of individuals and groups who are ‘suffering’ (A.E. v. Bulgaria, 2023; Alajos Kiss v. Hungary, 2010; Opuz v. Turkey, 2009), who are ‘victims’ of (domestic) violence or other harm and abuse (A.E. v. Bulgaria, 2023; Opuz v. Turkey, 2009), they are portrayed as having ‘an underdeveloped or weakened capacity to control their conduct’ (Khamtokhu and Aksenchik v. Russia, 2014), as fragile and dependent (A.E. v. Bulgaria, 2023) or as debased, hopeless and frightened (Opuz v. Turkey, 2009). In *Khamtokhu and Aksenchik v. Russia* (2014), the position of women as a ‘naturally vulnerable social group’ is discussed, in other cases the cause of vulnerability is described as being ‘a result of their turbulent history and constant uprooting’ (D.H. and Others v. The Czech Republic, 2007; Oršuš and Others v. Croatia, 2010). Furthermore, they frequently are associated with being ‘particular’ vulnerable, which cannot be ‘ignored’ or ‘overlooked’ (Enver Şahin v. Turkey, 2004; Çam v. Turkey, 2016), having ‘special needs’ (Khamtokhu and Aksenchik v. Russia, 2014; Konstantin Markin v. Russia, 2012; Oršuš and Others v. Croatia, 2010) and being in need of ‘special protection’ (A.E. v. Bulgaria, 2023; D.H. and Others v. The Czech Republic, 2007; Oršuš and Others v. Croatia, 2010). These narratives paint a picture of problematic, deficit-emphasizing, stereotypical and stigmatizing associations of people and groups who are considered vulnerable. They are those, who are characterized by suffering, neediness and particularities, who are not fully capable and dependent on protection. They rely on an implicit understanding of norm and deviation, the latter being the particularity of the vulnerable, victimized ‘other’.

The CJEU in contrast does not operate with the label of vulnerability in its extensive discrimination case law. Indeed, the awareness of the Court about the negative aspects of stereotyping has evolved over time. Recent case law makes clear that differences in the treatment of specific groups can only be argued for in very specific cases and that any risk of stereotyping should be avoided (Konstantinos Maïstrellis v Ypourgos Dikaiosynis, Diafaneias kai Anthropinon Dikaiomaton, 2015). While in the 1980s the court rulings still contained a stereotypical perspective on how families divide their work (Commission of the European Communities v Italian Republic, 1983), the last 15 years have seen an acknowledgment of equal roles and the right to equal treatment (Roca Álvarez v Sesa Start, 2010; Konstantinos Maïstrellis v Ypourgos Dikaiosynis, Diafaneias kai Anthropinon Dikaiomaton, 2015). Vulnerability is also not mentioned in the Court’s landmark cases on disability (Coleman v Attridge Law, 2008; Kalsten Kaltoft v Kommunernes Landsforening, 2014) or ethnic affiliation (“CHEZ Razpredelenie Bulgaria” AD v Komisia za zashtita ot diskriminatsia, 2015; Centrum voor gelijkheid van kansen en voor racismebestrijding v. Firma Feryn NY, 2008). The CJEU upholds the principle of EU equal treatment law that any legitimization of direct discrimination has to be interpreted very narrowly, which for our topic means that the situation of persons or groups of persons in a specific situation in most cases has to lack comparability to others in order to make a differentiation arguable. If we take this seriously, a reference to a presumed vulnerability of women, of persons with disabilities or a specific ethnic affiliation would not qualify as an argument in a CJEU case.

### 4.3 Relationship between vulnerability and equality/non-discrimination

In many cases, the identification of a person or groups of persons as being vulnerable or in a vulnerable situation has been used by ECtHR to support its conclusion that they had been affected by discrimination. However, when we look at the legal concepts (indirect discrimination, positive action measures and reasonable accommodation) that are available already, ‘the question remains in what way vulnerability […] could facilitate interventions or remedies to inequalities’ (Cole, 2016, p. 260) and if any eventual advantages are proportional to the risks of declaring whole groups as vulnerable and invoking the problematic narratives that are associated with the concept (see previous sub-section).

From an equality point of view, the application of the frame of vulnerability in these judgements is problematic as it means accepting unequal treatment on the grounds of sex, age, ethnic affiliation or disability based on the stereotypical assumptions of being more in need or requiring ‘special protection’ (Oršuš and Others v. Croatia, 2010, para. 147) or ‘being particularly careful’ (Çam v. Turkey, 2016, para. 66) simply on the grounds of membership to a certain group. This contradicts the Court’s principles that—at least for the ground of gender—any general and automatic restriction applied to a group of people based on their sex, irrespective of their personal situation, falls outside an acceptable margin of appreciation, however wide that margin might be, and consequently is incompatible with Article 14 (Konstantin Markin v. Russia, 2012, para. 148) and that women do not by default fall in the category of vulnerable persons (Valiulienė v. Lithuania, 2013).

With the understanding of the anti-discrimination regime as defined by the EU, which has been taken on board also by the ECtHR, a reference to the vulnerability of a person or groups of persons does not seem necessary—at least from a legal point of view. The principle of non-discrimination, with its acknowledgment of the concepts of reasonable accommodation and positive action measures, provides enough room to identify patterns of structural inequalities and call for specific measurements designed to reduce such inequalities. And—in contrast to the application of the vulnerability approach—the toolbox offered by the non-discrimination regime, entails less risks of being paternalizing, stigmatizing and victimizing.

The case study showed that the application of the concept of vulnerability is not necessary for a finding of discrimination. The concept is not defined in the legal framework, and the objectives behind its use are ambiguous. In the long run, the concept bears the risk of not reducing inequalities, but due to its stereotyping character increasing group think and discriminatory structures.

## 5 Case study 3: Vulnerability in Austrian decisions on international protection

The concept of vulnerability is widely used in the asylum context (Leboeuf, 2022, p. 2), also in the European context. At EU level, vulnerability has become part of the asylum acquis. Human rights courts such as the ECtHR or adjudication bodies increasingly use this concept in their reasoning (Leboeuf, 2022, p. 11). Given its widespread use in different contexts and stages of the asylum procedure, in particular since *MSS*,^15^ it has been described as ‘one of the central tools on which the every effectiveness of the international protection system hinges’, with a ‘decisive role’ regarding refugees’ and asylum-seekers’ rights (Yahyaoui Krivenko, 2022, p. 193). In ECtHR jurisprudence on the *non-refoulement* obligation under Article 3 ECHR ‘vulnerability’ would help in shifting focus on individual needs/power imbalances (e.g., Leboeuf, 2022, pp. 13–14) or contribute to the lowering of the very high threshold used in ‘medical cases’^16^ and cases of socio-economic deprivation (Flegar, 2016, pp. 160, 153). It was argued that by ‘identify[ing] socially disadvantaged groups in need of enhanced protection’ the Court would ‘focus attention on socially constructed patterns of power and disadvantage’ (Blöndal and Arnardóttir, 2019, p. 149). Yet, the case law analysed in this literature mostly does not use ‘vulnerability’ explicitly in the reasoning.^17^ Recently a ‘vulnerability backsliding’ at the ECtHR has been diagnosed (Hudson, 2024, focusing on asylum seekers’ vulnerability). Legal scholars have dedicated research in particular to the role of vulnerability in ECtHR jurisprudence with regard to asylum-seekers’ detention conditions or living conditions in European states (e.g., Yahyaoui Krivenko, 2022, p. 1) or to the potential of vulnerability as a conceptual tool for courts providing the ECtHR with recommendations (e.g., Heri, 2021; regarding ‘migratory vulnerability’ see Baumgärtel, 2019, 2020). Less research has been conducted on the role of ‘vulnerability’ in relation to eligibility criteria of international protection.^18^ Against this background, this case study sheds light on a national context. The case study focuses on jurisprudence of Austrian courts relating to decisions on international protection in which the concept of vulnerability was explicitly referred to in the legal reasoning.

At national level, in Austrian asylum procedures, the Austrian appellate court (BVwG) has increasingly employed the concept of vulnerability (in German: *Vulnerabilität*) in its assessments on international protection.^19^ Vulnerability has entered qualification criteria of international protection even though such criteria in Austrian asylum law or in the EU Qualification Directive 2011/95/EU do not contain this concept. Against the background as described in Section 2, this case study analyzed the application of the vulnerability concept in the case law of the BVwG and complementary in the case law of the Supreme Administrative Court (VwGH) and the Constitutional Court (VfGH). The section explores who the courts framed as vulnerable in asylum procedures, which sources of the applied concept of vulnerability were mentioned, which legal consequences courts attached to the usage of the concept and how the vulnerability concept contributes to a process of gendered racialization which is at odds with the principles of equality and non-discrimination. The case study is based on a quantitative and qualitative analysis of 394 decisions on international protection of the BVwG, containing explicit and rich engagement with ‘vulnerability’ in the legal reasoning,^20^ and supplemented by an analysis of all decisions of the VwGH and the VfGH as well as of interviews with legal stakeholders.^21^

Overall, vulnerability is featured more frequently in legal reasonings of the subsidiary protection assessment compared to legal reasonings relating to refugee status determination (RSD), where it is assessed whether a person fulfils the requirements of the refugee definition laid down in the Refugee Convention.^22^ In Austria, a real risk assessment (RRA) under Article 3 ECHR is conducted to determine whether subsidiary protection^23^ must be granted. The RRA requires the analysis of individual circumstances as well as general conditions in the receiving state (Vilvarajah and Others v United Kingdom, 1991; F.G. v Sweden, 2016; Blöndal and Arnardóttir, 2019; Directive 2011/95/EU, 2011). The VwGH demands a holistic assessment of risks (Ra 2018/18/0315, 2018a) in the context of which asylum authorities must take a ‘particular vulnerability’ of complainants into account (Ra 2018/18/0315, 2018a; Ra 2020/18/0165, 2022a). This in turn demands a concrete examination of the return situation that the complainants find at the place to be returned (Ra 2020/14/0096 to 0102, 2020). Also in the assessment of an internal protection alternative (IPA), individual circumstances as well as general conditions in the receiving state must be scrutinized (Directive 2011/95/EU, 2011, art. 8(2); Austrian Asylum Act, 2016, sec. 11(2)).

In the sample of BVwG decisions an unequal distribution of vulnerability with regard to gender and family was observable: While in decisions relating to men (57.4% of the total sample) and families (26.1% of the sample) vulnerability played a role mainly in the assessment of subsidiary protection, in decisions relating to women (only about 16.5% of the total sample) vulnerability featured mainly in legal reasonings regarding RSD. Also concerning the outcome of the proceedings in which vulnerability played a role in the legal reasonings—although not necessarily a decisive role—, considerable gender/family differences could be observed: While 55.3% of appeals by male applicants were dismissed, only 19.7% of appeals by female applicants and 29.1% by families were dismissed. The refugee status recognition rate was significantly higher in decisions concerning female applicants (58.5% compared to 11.5% of decisions relating to men and 12.6% to families). With regard to the granting of subsidiary protection, 49.5% of applications by families were successful compared to 16.9% women and 19.9% men.

### 5.1 Legal sources and definitions of the concept of vulnerability

Similar to the jurisprudence of the ECtHR, Austrian jurisprudence does not contain an explicit definition of vulnerability, even though it sometimes refers to existing definitions. The main source used by Austrian courts—including in the IPA assessment context—was article 21 of the EU Reception Conditions Directive 2013/33/EU (RCD). This provision obliges EU Member States to take ‘the specific situation of vulnerable persons’ into account in the national law implementing the RCD. It mentions in a non-exhaustive list groups of asylum seekers to be considered as ‘vulnerable persons’ with regard to reception conditions, who—if they also have special needs—are granted special treatment. Besides the fact that, from a legal perspective, the reference to article 21 RCD is not comprehensible since the objective of the RCD has nothing to do with eligibility criteria of international protection, two other aspects are important from an equality perspective: Firstly, the close relation of vulnerability with special needs. According to Art. 2(k) of the RCD only vulnerable persons can have ‘special’ reception needs. This, as in the two previous case studies, is based on an implicit hierarchical understanding of those whose needs are unmentioned as they are perceived as the norm and those whose needs are ‘special’ and require ‘special treatment’. Secondly, the ascription of the vulnerability concept to predefined groups. Both points might be understood to contribute to a gendered racialization of the legal discourse.

Other sources for (lacking) vulnerability mentioned by the courts—including in the IPA assessment—were UNHCR (UNHCR, 2019, p. 115) and/or EASO documents (E1524/2020, 2020; E1953/2020, 2020), also when arguing for instance that a single healthy man ‘without vulnerabilities’ could find a reasonable IPA without the existence of a social network (Ra 2017/19/0118, 2017). When referring to children as a vulnerable person or group, reference was made to the UN Convention on the Rights of the Child (CRC), article 24(2) of the EU Fundamental Rights Charter or national law (Bundesverfassungsgesetz über die Rechte von Kindern, 2011, art. 1), even though these sources do not explicitly refer to vulnerability but demand that the best interests of the child have to be a primary consideration. Austrian jurisprudence hardly referred to ECtHR jurisprudence as a source of its vulnerability reasoning, except to *Paposhvili v Belgium*, which, however, does not contain explicit ‘vulnerability’ in its reasoning.

### 5.2 Application of the concept: Who is framed as vulnerable? Narratives of vulnerability

In RSD, mostly single women, women ‘without male protection’ or ‘western-oriented’ single women were referred to as vulnerable (W211 1428338-1, 2014; W121 1416841-2, 2015; W108 2170868-1, 2021; W252 2226620-1, 2021). Men were labeled vulnerable only in exceptional cases (e.g., being an underaged member of a minority clan (W211 2118973-1, 2016)) in RSD. In the assessment of subsidiary protection, Austrian courts mainly regarded groups and persons as vulnerable. Exceptionally, persons were described to be in vulnerable situations or positions (L519 2208380-1, 2021; W183 2231964-1, 2021; I408 2221277-1, 2022), or certain conditions or characteristics (e.g., being pregnant) (E542/2020, 2020) were associated with vulnerability. In addition, the notion of ‘vulnerable to’ e.g. ill-treatment (W144 2219098-1, 2020; W144 2201411-1, 2021) or discrimination (W229 2189239-1, 2021) was used. The VwGH and VfGH primarily identified children (E2526/2019, 2019; E1524/2020, 2020; E3524/2019, 2020), families with children (E2047/2021, 2022), pregnant women (E428/2018, 2019; Ra 2019/18/0187, 2019); persons with health issues and/or disabilities (U496/2013, 2013; E3796/2018, 2019; Ra 2020/18/0064, 2021) as vulnerable. Both courts require to consider also multiple ‘vulnerabilities’ (E1524/2020, 2020; E1689/2020, 2020; E1953/2020, 2020; Ra 2020/18/0056, 2020; Ra 2019/18/0451, 2021). This case law of VwGH and VfGH is reflected in the case law of the BVwG (L519 2165668-1, 2022). However, courts also described vulnerability as a result of certain infrastructural conditions: e.g. the VfGH deduced a particular vulnerability from the distance to possible health treatment, limited mobility and lack of infrastructure for wheelchair users in Armenia (E4491/2021, 2022a). The BVwG mentioned as causes of vulnerability, e.g., a ‘high-risk pregnancy’ (W204 2187556-1, 2020), (mental) health issues, age and traumatization (I403 2111449-1, 2015), being single (W125 1433061-1, 2014a), gender, minority status; having care obligations, poverty (I403 2014721-1, 2016), lack of education or work experience; inability to work; lack of a support network (W125 1433061-1, 2014b; I403 2111449-1, 2015; L516 2160087-1, 2016), or long absence from the country of origin (W206 1418588-2, 2014). Sometimes the Court referred to several causes at the same time (W125 1433061-1, 2014a; W206 1418588-2, 2014; I403 2111449-1, 2015; I403 2014721-1, 2016). If persons or groups were described as vulnerable, overall, the focus seemed to be on ‘visible’ features, e.g., related to age, physical impairments/disabilities, pregnancy, or gender. Such as focus could be regarded as critical with regard to the concept of racialization (see Section 2).

In many cases relating to the assessment of subsidiary protection concerning male applicants, a lack of vulnerability was indicated, both in the RRA and the assessment of an IPA. Single, healthy, adult males ‘without particular vulnerability’ were regarded to be in a position to use an IPA in Afghanistan even if they lacked a social network (W245 2136893-1, 2017). Vulnerability was also sometimes compared and finally denied, e.g., when the BVwG argued that a 17-years-old child was not more vulnerable than an 18-years-old adult (W122 2205567-1, 2018; W270 2159621-1, 2018). The ‘absence of vulnerability’ was regarded to be proven because—among other things—a complainant was able to undertake the dangerous journey to Europe, manage the ‘enormous psychological tension of the uncertainty of what could await him in Europe’ and organize his life also after impairments due to a suicide attack (W105 2161679-1, 2020).^24^ Besides the obvious gendered assignment of the concept of vulnerability in the examples above, it is also striking that the concept is mainly used with regard to individual circumstances and (in)abilities. This is in contrast to concepts of discrimination which focus on the unequal treatment of different persons or the unequal effect of practices or norms on different individuals and groups.

### 5.3 Modest legal consequences of the vulnerability concept

The VwGH obliges asylum authorities to conduct a holistic assessment of possible risks from the point of view of particular vulnerability, in view of the special need for protection (E4491/2021, 2022b; Ra 2020/18/0185 to 0188, 2022). Asylum authorities must draw conclusions from this vulnerability and assess in detail the concrete return situation for the individual or group. Concrete findings in this context are necessary (Ra 2019/18/0368, 2020; Ra 2021/18/0349, 2020; Ra 2017/18/0474 to 0479, 2018), unsubstantiated assumptions (e.g., on the existence of a family network providing support) are not enough (Ra 2018/18/0315, 2018b; Ra 2020/18/0165, 2022b; Ra 2020/18/0185 to 0188, 2022). Up-to-date and relevant COI reflecting experiences of particularly vulnerable applicants, e.g., child-specific COI, is required (E149/2021, 2021). The BVwG reflects these requirements of the VwGH and VfGH in its jurisprudence.

Yet, several of these legal requirements resulting from the concept of vulnerability are not new obligations. Already before this jurisprudence, asylum authorities were required to evaluate—based on relevant COI—the concrete return situation and to take individual circumstances into account.

### 5.4 Relationship between vulnerability and equality/non-discrimination

The analysis reveals that some elements that are important in assessing cases of discrimination or inequality are also relevant in vulnerability assessments. For example, turning to decisions relating to subsidiary protection, there is an inherent element of comparison in the non-refoulement assessment: According to the jurisprudence of the ECtHR, the applicant usually must provide evidence of ‘special distinguishing features’ showing a personal and individualized risk of ill-treatment (Blöndal and Arnardóttir, 2019); demonstrating that one would be in a worse position than ‘the generality of’ people in a similar situation (Vilvarajah and Others v United Kingdom, 1991, para. 111). It is in this context (mainly) of individual circumstances that Austrian courts usually refer to vulnerability. Based on our review, Austrian courts used the concept of vulnerability often to argue that certain persons or groups appeared to be more or less at risk of ill-treatment than others or that certain characteristics were regarded to make a person more or less prone to ill-treatment. While vulnerability was never the sole argument for or against granting protection, its usage was not always accompanied by a detailed explanation of why the complainant was deemed to be more or less at risk than others. However, vulnerability does not relieve the requirement of an individual assessment of each case.

With regard to the question of whether the concept of vulnerability leads to an enhancement of equality in Austrian decisions on international protection, the analysis of the case law suggests that the concept has the potential to contribute to a gendered and racialized representation of asylum seekers which is at odds with principles of equality and non-discrimination. As already indicated in Section 2, the term racialization is a concept which aims at grasping ‘the process through which *racialized* groups […] are formed’ (Hochman, 2019, p. 1245). It ‘refers to a process of categorisation, a representational process of defining the Other, usually, but not exclusively, somatically’ (Miles and Brown, 2003, p. 101). Firstly, vulnerability is a somatic metaphor which is used as a marker to distinguish between those who are eligible for international protection (certain vulnerable groups and persons) and those who are not eligible for international protection (‘lack of vulnerability’). As the analysis above showed, vulnerability as a somatic issue was frequently used by the courts to categorize individuals and groups on basis of phenotypical features (e.g., sex, age, impairments/disabilities, pregnancy). Secondly, the case law reveals that there is a striking gender difference in the outcome of the decisions with vulnerability reasoning (see also quantitative data at the beginning of this section). The cases distinguish in particular between the vulnerability of single women ‘without male protection’ who were often granted a refugee status (‘a single woman […] is a particularly vulnerable person in need of protection’ (L519 2208380-1, 2021), ‘due to the already increased vulnerability per se as a woman’ (W127 1431444-3, 2012), ‘a particular vulnerability for single female minority members’ (W127 1431444-3, 2012)) and the lack of vulnerability of single men (‘a single, healthy and able-bodied man is one of the least vulnerable people’ (G307 2183119-1, 2021)). Thus, women were repeatedly marked as vulnerable in need of protection while men, in particular single, able-bodied, healthy, young men were marked as lacking vulnerability or not being vulnerable enough to deserve international protection. This contributes to a stereotypical representation of masculinity and femininity. Thirdly and closely connected with the last point, the case law shows explicit examples that depict a racialized portrayal of a traditional, intolerant culture which exploits and abuses vulnerable persons and groups,^25^ in particular ‘Western-oriented’ women (W127 1321444-3, 2014; W159 2110761-1, 2017; G304 2195776-1, 2018; W158 2173356-1, 2018; W144 2183564-1, 2019). This is contrasted with a tolerant Western society where a woman who ‘has adopted a Western way of life […] enjoys her freedom and her right to self-determination’ (G304 2195776-1, 2018). Besides the fact, that these points are problematic issues with regard to the enhancement of equality, furthermore, the analysis suggests that the concept of vulnerability is often attached to certain pre-determined identity-based groups who are represented as ‘special’ and deviant from a certain norm as they have special needs, need special treatment and special protection.

## 6 Comparative discussion and conclusions

This article set out to explore the usage of the concept of vulnerability in three case studies. The three case studies focus on different thematic areas (climate change and mobility in UN human rights documents, anti-discrimination at European level in particular in case law of European courts, Austrian decisions on international protection) and different levels (global, regional and national) and used different materials and methods (document analysis, case law analysis, qualitative interviews with stakeholders). Yet, the case studies also come to similar observations and conclusions with regard to the application of the concept of vulnerability.

First of all and as pointed out by the literature discussed in Section 2, the question how vulnerability is defined is challenging in all three case studies. Vulnerability proves to be a broad, fuzzy, ambiguous and elusive concept (Brown et al., 2017, p. 505; Chapman and Carbonetti, 2011, p. 723, Kim, 2021, pp. 625–626) in all three analyzed cases. In the case study on climate change and mobility in UN human rights documents, vulnerability is used predominately without providing a clear-cut and unambiguous definition. When a definition is provided, it rather focuses on individual (in)abilities and not on unequal treatment and unequal effects of practices and structures on individuals. Vulnerability is also neither mentioned in the legal framework nor defined in policy documents relevant to the case study focusing on anti-discriminaiton case law of European courts. Also in this case study, we can see that the vulnerability concept shows the tendency to frame the persons labeled with this concept as the problem, as it is ‘their needs’, ‘their different lifestyle’, ‘their history’ or ‘their position’ which is referred to in the context of the vulnerability reasoning. With regard to the third case study, it was found that Austrian jurisprudence in the field of asylum law does not contain a definition of vulnerability, although it sometimes refers to EU asylum law, in particular Article 21 RCD or UNHCR documents. The RCD definition relates the vulnerability concept not only to special needs but also to a predefined, non-exhaustive list of groups. In addition, also in the asylum case law the concept is mainly used with regard to individual circumstances and (in)abilities. As already indicated in the case studies, this marks a striking difference to concepts of discrimination which focus on unequal treatment or unequal effect of practices or norms on different individuals and groups.

Secondly, in all three case studies the objectives, motivations, and added value for introducing and using the concept are often unclear, which substantiates the scholarly critique of the ambiguity, elusiveness and fuzziness of the concept (see Section 2 and previous paragraph). The point of using the term seems to be to emphasize the urgency of the matter, to evoke compassion, or to support the argument that a certain case needs special attention or treatment. In the case study on climate change and mobility in UN human rights documents, usually, a broad range of problems associated with climate mobility are listed and persons who are assumed to be confronted with these problems are labeled as vulnerable. The framing of affected groups, individuals, communities or situations as vulnerable would not be necessary as the problem could also be described differently. With regard to the case study on anti-discrimination case law in European courts, a reference to the vulnerability of persons or groups also does not seem necessary from a legal point of view as the European legal framework provides appropriate concepts and measures in order to identify patterns of structural discrimination. In contrast to the ECtHR, the CJEU does not use it. Also, in Austrian decisions on international protection the concept seems to have no or only modest added legal value as the concept of vulnerability does not lead to major new legal requirements. Furthermore, it could not be established that the motivation and objective for introducing the concept is the promise that it enables a more thorough and pervasive equality analysis as suggested by Fineman (2008, p. 17).

Thirdly, in all three case studies the concept is frequently applied to specific groups. It is not apparent in the case studies that the concept is detached from identity categories and that it provides for a ‘post-identity’ inquiry of inequalities as suggested by Fineman (2008, p. 16). In most of the cases, no objective and comprehensible criteria are discernible or defined for deciding who is classified as vulnerable. Although there is a considerable variety of groups as well as sub-groups labeled as vulnerable in UN documents on climate change and mobility, some groups (women and sub-groups of women, children and people living in poverty) are identified more often to be vulnerable than others. There is also a lack of guidance on the criteria of who is classified as vulnerable in the case study on anti-discrimination case law of European courts. In the case law of the ECtHR, vulnerability is predominantly applied in Roma and disability cases and sometimes also in cases referring to women. In the case law analyzed for the third case study mostly single women, children, families with children, pregnant women, persons with health issues and/or disabilities were framed as vulnerable. A lack of vulnerability was frequently mentioned with regard to single, healthy, adult men. The analysis in this case study showed that a focus is often laid on phenotypical characteristics of persons and that there is an unequal, gendered distribution of vulnerability, which, when taking into account that vulnerability also constitutes a somatic metaphor, may contribute to a gendered racialization of the legal discourse (see ECRI, 2021, para 3; see also Urquiza Haas and Sánchez García, 2015, p. 152).

Fourthly, in all three case studies the mobilization of problematic and/or stereotypical narratives could be observed in association with groups and individuals labeled as vulnerable, which substantiate a frequent and well-elaborated point of scholarly criticism (Brown, 2011, 2017; Kadetz and Mock, 2018; Peroni and Timmer, 2013; Scully, 2009; Urquiza Haas and Sánchez García, 2015; Wisner, 2004, p. 13). UN human rights documents focusing on climate mobility often contain a broad range of harm, abuses, risk and sufferings associated with vulnerable persons and groups. Vulnerabilities are frequently phrased as a failure or flaw of the specific person or group and ‘implying deviation from usually undefined standards of life or behaviour’ (Brown et al., 2017, p. 498). They are framed as persons and groups with special or particular needs who require protection and particular or priority attention (see also Smith and Waite, 2018, p. 1; Howden and Kodalak, 2018; Yahyaoui Krivenko, 2022). The latter is also discernible in ECtHR discrimination case law as well as in Austrian case law on international protection. However, the case study on asylum also revealed that vulnerability was not always associated with only one single, but with several characteristics or circumstances of a person. There is also some awareness of involved stakeholders that the concept of vulnerability might contribute to a stereotypical representation of individuals and groups, which is either expressed explicitly in the analyzed documents (first and second case study) or which was voiced during interviews by stakeholders (second and third case study).

That leads to the fifth point, the relation of the concept to the concept of equality and non-discrimination. The material analyzed (case law, policy documents) suggests that vulnerability is understood by the authors of these materials (courts, policy makers or other relevant actors) as in some way interconnected with discrimination and inequality. In the first case study, the documents reveal that the authors of the analyzed documents assume that inequality and discrimination lead to vulnerability and this in return results in increased inequality and discrimination. In the second case study, the identification of persons and groups as vulnerable has helped the court to support a finding of discrimination and in the third case study, the fear of discrimination in the country of origin of the asylum seeker has been marked as a particular vulnerability in some cases. This indicates that vulnerability is understood as something that is negatively connected to the right to equality and non-discrimination or even hampers equality and non-discrimination, which was also argued by other scholars (see, e.g., Brown, 2017; Cole, 2016; Ferrarese, 2016; Scully, 2009). Thus, the label is not assumed to have empowering and equality-promoting connotations by the authors of the material examined in all three case studies. This has, however, not led to an abandonment of the concept as such, although some doubts have been raised—as was indicated in the previous paragraph—that involved stakeholders are to some extent aware that the use of the concept itself is a problematic practice which has adverse consequences with regard to the principles of equality and non-discrimination. These problematic practices and their consequences could also be observed in the three case studies. Vulnerability is a loaded and value-laden concept and refers, as already indicated, to a somatic metaphor, which contributes to sexist, ableist, racist and other stereotypical narratives and representations. It contributes to the racialization of the legal and political discourse (see ECRI, 2021), which is highly problematic with regard to the right to equality. As mentioned above, covert racist and also sexist discourses and language often deploy narratives and metaphors that imply in- and exclusion (vulnerable—not vulnerable), negative associations, stereotypical representations and label specific persons and groups as ‘somatically’ different. The metaphor of vulnerability (wound and woundedness) implies these associations. Yet, as indicated in Section 2, stereotypical narratives and representations are discriminatory and reinforce structural discrimination and inequalities and, thus, they are prohibited by human rights law. Vulnerability reasoning does not focus on structural factors (see also, Cole, 2016, p. 266; Brown, 2017, pp. 674–675 and Sections 3.2, 4.2 and 5.2), it rather puts a label or marker on individuals and groups and frames ‘individual circumstances’ as their vulnerability.

From our case studies, we can conclude that the concept of vulnerability does not live up to the promises indicated by proponents of the concept outlined in Section 2 of this article. Instead, it rather confirms the critical points raised by many scholars concerning the concept. The case studies corroborate that vulnerability, in practice, is hardly ever clearly defined, that the concept yet again gets stuck with group-based identity categories, it has problematic effects on people excluded from the concept (in particular in the asylum context) and the concept is stigmatizing and mobilizes racialized, sexist and other discriminatory narratives, which are in conflict with important criteria of substantive equality. It strengthens individual deficit accounts of disadvantage and fails to grasp and tackle structural factors of discrimination and inequality. As also apparent in the case studies, vulnerability approaches are often associated with giving ‘special attention’ or ‘special protection’ to those (groups of) persons who are in ‘particular need’ and, thus, presenting them as a deviation from an undefined norm. However, and as was also observable in the case studies, these approaches often fail to conform with criteria, laid down by international law for proactive measures in order not to be considered as discrimination (see Section 2), such as being objective-oriented and evidence-based or not enhancing stereotypes and stigmatization.

To enhance the protection of human rights it is important to pursue a human rights-based approach, which understands human beings as rights-holders and aims at realizing human rights of people based on international human rights standards rather than addressing ‘the needs of beneficiaries’ (UNFPA, 2014). Thus, it is important to consider the following concluding points concerning the concept of vulnerability: Firstly, as vulnerability is not defined or even mentioned by human rights instruments, it is recommended to rely instead on codified and well-developed human rights standards, in particular on the rights to equality and non-discrimination. Non-discrimination laws and policies are characterized by an elaborated toolset of definitions and concepts to describe and, respectively, address discrimination and inequality. These definitions and concepts are constantly further developed, as, for example, the *General comment No. 6 (2018) on equality and non-discrimination* published by the Committee on the Rights of Persons with Disabilities shows. Secondly, it is essential to evaluate if concepts which are used in legal and political processes have an added value that contributes to advancing equality and non-discrimination. Thirdly, to avoid the problematic consequences of group-based approaches, which are often associated with the concept of vulnerability (i.e., vulnerable groups), it is better to focus on grounds or categories of discrimination and inequality (such as gender, age, race) instead of groups. This is already provided for in most international and regional human rights frameworks—however, also often incorrectly applied. Fourthly, it is important to be aware of the legal obligations with regard to addressing any form of stereotypical language and prejudices against persons and groups and of the risks the vulnerability approach constitutes in this regard. Fifthly, proactive measures to enhance equality are important. Yet, it is also crucial that such proactive measures comply with criteria, which are laid down in international or regional human rights documents, in order not to be considered unlawful discrimination.

## Data Availability

The original contributions presented in the study are included in the article, further inquiries can be directed to the corresponding author.

## Footnotes

^1^In doing so, the research adopted a comparative design of multiple case studies with most-different cases [for the logic of such a research design see, for example, Anckar (2020) or Clark et al. (2021)].

^2^With the exception of the third case study, where also – to a very limited extent – a quantitative analysis was carried out.

^3^This was done by using the qualitative data analysis program MAXQDA.

^4^Concerning the regional levels, this section will focus on the European regional system as this is relevant for the case studies presented below.

^5^The roots of which go back much further than the first codification of a global human rights treaty (see, for example, Pilcher and Whelehan, 2004; Moeckli, 2018, p. 149).

^6^This is laid down in many international human rights instruments and documents (see, for example, International Covenant on Civil and Political Rights, 1966, art. 26; Human Rights Committee, 1989; Committee on Economic, Social and Cultural Rights, 2009; Committee on the Rights of Persons with Disabilities, 2018) [henceforth CRPD GC 6].

^7^This is confirmed not only by academic literature but also by documents of different political and judicial bodies (see, for example, Moeckli, 2018; Otto, 2018; European Commission, Directorate General for Justice and Consumers et al., 2022; Committee on Economic, Social and Cultural Rights, 2009; Human Rights Committee, 1989; Committee on the Rights of Persons with Disabilities, 2018; Committee on the Elimination of Racial Discrimination, 2005).

^8^This is not only laid down in international human rights law but was also emphasized in many documents as well as by academic literature (see, e.g., Convention on the Elimination of All Forms of Discrimination Against Women, 1979, art. 5; Convention on the Rights of Persons with Disabilities, 2006, art. 8; Committee on the Elimination of Racial Discrimination, 2005; Committee on Economic, Social and Cultural Rights, 2009; CEDAW Committee, 2010; European Commission against Racism and Intolerance, 2015; Committee on the Rights of Persons with Disabilities, 2018; Fredman, 2016b).

^9^UN treaty monitoring bodies as well as regional courts and organizations have developed such criteria (see, for example, CEDAW Committee, 2004; CERD Committee, 2009; Committee on the Rights of Persons with Disabilities, 2018; European Commission, Directorate-General for Employment, Social Affaires and Equal Opportunities, 2009; Equinet, 2014).

^10^This also means, that state and other reports submitted in the context of reporting procedures such as the Universal Periodic Review or specialized human rights treaties, were not considered in this study.

^11^Some documents indicate, that there is an ongoing discussion on whether the concept of vulnerability is contributing to the stereotypical representation of individuals and groups. E.g., the CEDAW Committee warns that ‘[t]he categorization of women and girls as passive “vulnerable groups” in need of protection […] is a negative stereotype’, which neglects ‘to recognise the important contributions of women’ (Committee on Economic, Social and Cultural Rights, 2018).

^12^Interviews referred to in this section were conducted online between May and July 2021.

^13^There is a considerable range of other ECtHR case law (e.g., asylum cases) that applies vulnerability terminology, however, cases that do not use the vulnerability concept in relation to discrimination claims (Art. 14 ECtHR) fall outside the scope of this case study.

^14^The ECHR and the its interpretation by the ECtHR are highly relevant for the EU anti-discrimination acquis as the CFREU in its respective article 52/3 includes a reference to the meaning and the scope of the corresponding rights in the ECHR as well as its interpretation by the ECtHR as well as the obligation to ensure a consistent interpretation.

^15^European Court of Human Rights (ECtHR), *M.S.S. v. Belgium and Greece*, Judgment, Grand Chamber, Appl. No. 30696/09, 21 Jan. 2011, para. 232.

^16^Cases in which migrants fight expulsion in order to continue to receive medical treatment in the sending State, e.g., ECtHR, *N v. United Kingdom,* Judgment, Grand Chamber, Appl. No. 26565/05, 27 May 2008.

^17^E.g. Flegar (2016) at p. 154 confirms this regarding ‘exceptional circumstances’ and *D v. United Kingdom*. Leboeuf argued that in *SHH v. United Kingdom* (Judgment, Appl. No. 60367/10, 29 Jan. 2013), the ECtHR focused *inter alia* on the applicant’s vulnerable position as a disabled individual, even though ‘vulnerability’ did not form part of the Court’s core reasoning relating to Art. 3 (Leboeuf, 2022, p. 6). Heri noted ‘a real shift of the balance in applicants’ favour – and in the favour of consistent vulnerability reasoning’ with *Paposhvili* (ECtHR, *Paposhvili v. Belgium*, Appl. No. 41738/10, Judgment, Grand Chamber, 13 Dec. 2016, paras. 182–83)—even though the ECtHR had not employed ‘vulnerability language’ in its legal reasoning. Heri explains this with ‘a certain degree of reluctance to set an overly broad precedent in this type of case’ (Heri, 2021, p. 101).

^18^Authors at least thematizing this topic are, e.g., Leboeuf (2022); Heri (2021); Flegar (2016); Brandl and Czech (2015).

^19^The share of decisions on international protection of the sample containing vulnerability has been increasing since 2014 (up to 16.2% in 2020).

^20^More than 24,400 decisions of the BVwG contained vulnerability (out of 87,064). Quantitative text analysis of court decisions available in Austrian legal database (RIS) 01.01.2014–01.11.2022 (search date: 8.11.2022): 8,176 decisions contained vulnerability in the section on legal reasoning.

^21^In total, 17 semi-structured interviews were carried out in August, September and October 2021.

^22^Vulnerability featured in 25% of legal reasoning relating to RSD versus 42% of legal reasoning relating to subsidiary protection assessment.

^23^Subsidiary protection is a legal status defined by Directive 2011/95/EU of the European Parliament and of the Council of 13 December 2011 on standards for the qualification of third-country nationals or stateless persons as beneficiaries of international protection, for a uniform status for refugees or for persons eligible for subsidiary protection, and for the content of the protection granted (recast) 2011, QD [henceforth Directive 2011/95/EU].

^24^This contrasts with the case law of the ECtHR which, e.g., in *M.S.S*. regards migratory experience (‘everything he had been through during his migration’) as a source of vulnerability of asylum seekers (*M.S.S. v Belgium and Greece* [GC] App no 30696/09 (ECtHR, 21 January 2011), para. 232). Compare Hudson (2018, p. 37).

^25^For example, in W144 2201411-1 it is said that ‘Afghan society generally has a very low tolerance towards people who are perceived as insulting or rejecting Islam, and that people who allegedly violate religious and social norms are vulnerable to mistreatment […].’ (W144 2201411-1, 2021).
